# Bush animal attacks: management of complex injuries in a resource-limited setting

**DOI:** 10.1186/1749-7922-6-43

**Published:** 2011-12-22

**Authors:** Katrina B Mitchell, Vihar R Kotecha, Alphonce Chandika

**Affiliations:** 1 Weill Cornell Medical College 1300 York Avenue, New York NY 10065-4896, USA; 2 Weill Bugando University College of Health Sciences, P.O.Box 1464, Mwanza, Tanzania

## Abstract

**Introduction:**

Though animal-related injuries and fatalities have been documented throughout the world, the variety of attacks by wild animals native to rural East Africa are less commonly described. Given the proximity of our northwestern Tanzania hospital to Lake Victoria, Lake Tanganyika, and the Serengeti National Park, and presentation of several patients attacked by bush animals and suffering a variety of complex injuries, we sought to report the pattern of attacks and surgical management in a resource-limited setting.

**Materials and methods:**

Four patients who were admitted to the northwestern Tanzania tertiary referral hospital, Bugando Medical Centre (BMC), in 2010-2011 suffered attacks by different bush animals: hyena, elephant, crocodile, and vervet monkey. These patients were triaged as trauma patients in the Casualty Ward, then admitted for inpatient monitoring and treatment. Their outcomes were followed to discharge.

**Results:**

The age and gender of the patients attacked was variable, though all but the pediatric patient were participating in food gathering or guarding activities in rural locations at the time of the attacks. All patients required surgical management of their injuries, which included debridement and closure of wounds, chest tube insertion, amputation, and external fixation of an extremity fracture. All patients survived and were discharged home.

**Discussion:**

Though human injuries secondary to encounters with undomesticated animals such as cows, moose, and camel are reported, they often are indirect traumas resulting from road traffic collisions. Snake attacks are well documented and common. However, this series of unique bush animal attacks describes the initial and surgical management of human injuries in the resource-limited setting of the developing world.

**Conclusion:**

Animal attacks are common throughout the world, but their pattern may vary in Africa throughout jungle and bush environmental settings. It is important to understand the management of these attacks in resource-limited health care environment. Further, the growing population and human encroachment on previously wild habitats such as the northwestern Tanzania bush argues for increased community awareness to assist in prevention of human injuries by animals.

## Introduction

Human injury resulting from encounters with non-domesticated animals is increasingly common throughout the world, particularly as ecosystems change and humans encroach on previously wild land [1]. Though the management of injuries resulting from dog bites, zoo animal attacks, and trampling or kicking by large mammals such as cows, moose, or deer, is facilitated by well-developed emergency response systems in the western world [2], more unusual wild animal attacks and the complex injuries that result may pose a challenge to surgeons practicing in resource-limited settings. Further, many reports of these attacks in Africa are drawn from the lay press and associated with tourist activity, and much less is written to guide management of injuries suffered by local populations during activities of daily living [3].

Given the populous nature of bush animals throughout the rural northwestern Tanzania region of Lake Victoria, Lake Tanganyika, and the Serengeti National Park, and the increasingly frequent human encounters with them, there is new need to document attacks, patient management, and outcomes. While local health care systems may be familiar with triaging common dog and snake bites, guidance regarding the management of larger and more unusual bush animals is lacking. We believe that utilizing basic trauma survey principles, infection control, and necessary surgical management can provide appropriate outcomes in resource-limited settings.

## Materials and methods

Four patients with wild animal attacks who presented in 2010-11 to the northwestern Tanzania tertiary referral hospital, Bugando Medical Centre (BMC), were documented. Though dog and snake bites commonly are managed on both an in- and outpatient basis at BMC, these four unusual animal attacks included a vervet monkey, hyena, crocodile, and elephant and resulted in complex injuries all requiring extensive surgical decision-making and repair. They represented particularly challenging cases unique from those seen with dog and snake bites. The patients ranged in age from six to 42, and all but one was participating in food-gathering or guarding activity at the time. Given the type and variety of animals involved in the attacks, and the potential for future attacks in a setting of increasing proximity of humans to wild animal natural habitat, the management and outcomes of these remarkable cases were documented to guide future treatment of similar cases. Common themes of tetanus, rabies, and antibiotic treatment for all patients were emphasized.

## Case Presentations/Results

### Vervet Monkey

A 6-year-old male was attacked by a vervet monkey while playing outside in a rural village. The monkey primarily attacked his face, tearing the soft tissue of his right cheek and mandibular area and exposing his teeth. The patient presented to an outside hospital, where the wounds were cleaned and pressure applied for hemostasis. He was transferred to the Casualty Ward of our hospital six hours after injury, where a trauma survey revealed no other injuries. His vital signs were normal. He received intravenous ceftriaxone and metronidazole. On the surgical ward, he received tetanus toxoid and rabies post-exposure prophylaxis. His wound was cleaned and dressed with moist gauze. Given the large amount of soft tissue loss suffered in the injury and the difficulty in performing a flap coverage operation in our resource-limited setting, the decision was made to allow the patient to granulate his wounds. When adequate granulation was achieved after two months, the patient was taken to the operating theatre for reconstruction of his upper lip wound. Partial closure was achieved. However, the patient did regain the ability to chew and swallow his food; his ability to control saliva remained partially impaired. He maintained appropriate nutrition and has suffered no other complications of his attack or unrelated illnesses. He will be referred to a specialist center for definitive closure and reconstruction by plastic surgery.

### Hyena

A 27-year-old female who was retrieving water in her semi-rural village suffered an unprovoked attack by a hyena. Given the relative proximity of her village to Mwanza City, she was brought to our Casualty Ward four hours after her attack, where trauma survey revealed only soft tissue injuries to her face, left hand, and left elbow region. She was hemodynamically normal. She was admitted to the surgery ward and administered intravenous metronidazole and ceftriaxone, tetanus toxoid, and rabies post-exposure prophylaxis. Unlike the pediatric patient, this female patient suffered only disruption of skin lines and no loss of soft tissue. Given the high risk of infection in a hyena bite and unknown underlying immunologic status of the patient, the wounds were cleaned vigorously with betadine and kept open for three days with moist gauze for protection. When no evidence of infection developed, she was taken to the operating theatre for delayed primary repair of her skin. The patient had an uneventful recovery and was discharged one week post-operatively.

### Crocodile

A 40-year-old male was fishing on a small handmade boat in Lake Tanganyika when a crocodile attacked and pulled him into the water. The crocodile partially swallowed the patient, crushing his left forearm and biting his chest and right shoulder region. The patient used his fishing knife to stab the crocodile and break free from its grasp. His family members rescued him from the water and transported him to a district hospital. When the district hospital determined that the patient needed surgery for his left upper extremity, they transferred him one day later to BMC. On trauma survey at BMC, the patient was tachycardiac to 110 beats per minute, hypotensive to 100/60 mmHg, and tachypneic to 35. Pulse oximetry was not available. He had dullness to percussion and decreased breath sounds on the left side. He had an obviously deformed crush injury of his left forearm with devitalized tissue, and his right shoulder had superficial to deep bite marks. His abdomen was non-tender. His hemoglobin level was 7.0. A chest x-ray showed left lung field homogenous opacity and four broken ribs.

A left chest tube returned 400 cc of fresh red blood. He received 2 liters of normal saline and one unit of whole blood through a large bore peripheral intravenous line. He was administered intravenous metronidazole, ceftriaxone, and tetanus vaccine. He was taken to the operating theatre the day of his admission to BMC for debridement of his left forearm and exam under anesthesia of his chest and right shoulder wounds.

His wounds were irrigated with betadine and left open for three days; he then returned to the operating theatre for delayed primary closure of his right shoulder wound and further debridement of his left forearm wound. His chest tube was removed one week after injury. Given the extensive crush injury to his left extremity and neurovascular disruption, the patient was taken to the operating theatre three week after injury for completion amputation. His recovery was uneventful and he was discharged home two weeks post-operatively.

### Elephant

A 43-year-old man was guarding his crops when a group of elephants entered his farm. One elephant attacked him from behind, hitting him with its trunk and trampling with its feet, then lifting and dropping him from the air with its tusks. Due to his rural location and poor emergency transport to a tertiary care center at the time of the attack, the patient presented first to a district hospital and several hours later was transported to our Casualty Ward. On arrival, the patient denied loss of consciousness but described shortness of breath and pain in his left leg. The patient was alert and oriented but dyspneic. He was tachycardic to 120 and his blood pressure was 130/80. His respiratory rate was 30 and pulse oximetry was not available for measurement. His chest was dull to percussion bilaterally, and he had decreased breath sounds bilaterally. His abdomen was non-tender. He had a closed but deformed left lower extremity below the knee. Pulses were intact and his foot was warm. Hemoglobin was 8.0. Chest x-ray showed homogeneous left chest opacity suggestive of hemothorax with nine broken ribs; his right chest had one broken rib. A tibia-fibula x-ray showed a comminuted tibia-fibula fracture. The patient was given 2 liters of normal saline and one unit of packed red blood cells through a large bore peripheral intravenous line. A left chest tube was placed and returned 500 cc fresh blood. The patient was taken to the operating theatre for placement of an external fixation device for his leg fracture. The chest tube was removed hospital day five and the external fixator removed two months later and he was non-weight bearing until this time.

## Discussion

The rural African experience differs from those injuries reported in more urban or developed areas of the world, where injuries secondary to animals often are from semi-domesticated farm animals or a result of motor traffic collisions rather than direct attacks [4,5]. Other wild animal attacks commonly reported from the developed world are those occurring in zoos or animal sanctuaries [6].

It is widely acknowledged that the growing human population in Africa has brought animals and humans into closer physical contact, and prompted higher rates of animal attacks on humans [7]. This appears increased during times of drought and decreased availability of crop food, as well as when humans venture off frequently used paths [8]. It also is known that vervet monkeys and hyenas are living in close contact to human beings in rural East Africa, and humans are moving ever closer to the previously protected ecosystems of the elephant in Northwestern Tanzania [9,10]. While best documented in the Australian literature, human encounters with crocodiles--particularly in lake regions of southeast Africa--have also been described [11].

Though our cases describe all direct animal to human attacks, the bush animals responsible for the attacks and their pattern of inflicting injury varied. Large cats and dogs attacking humans have demonstrated that they attack the face and neck region of their victims, attempting to cause submission of their prey by damaging the cervical spine region [12,13]. The hyena, which resembles a dog but genetically is similar to a cat, followed this pattern in attacking our female patient. Injuries and deaths resulting from encounters with elephants most commonly result from trampling and less commonly secondary to a penetrating tusk stab wound [14]. Unlike other animals that often only attack humans when their nesting or feeding area is threatened, crocodiles are considered "opportunistic feeders" that may attack unprovoked. They usually crush victims with their powerful jaws [15]. Though there are scarce reports of vervet monkey patterns of attack documented in the literature, it has been shown that other primates such as chimpanzees attack more frequently based on scarcity of native food related to changing weather patterns [16,17].

Aside from causing internal organ injury and soft tissue damage, animal attacks also may transmit infectious diseases. Vervet monkeys have been shown to carry multiple parasitic and bacterial diseases, as well as viruses transmissible to humans [18]. These include Rabies, Ebola Reston, Herpes B Virus, Monkeypox, Yellow Fever, Simian Immunodeficiency Virus, and tuberculosis [19]. Rabies is the most commonly acknowledged disease transmitted from cats or dogs to humans, and this extends to hyenas [8]. Crocodile mouths may harbor *Aeromonas hydrophilia*, *Pseudomonas aeruginosa*, *Proteus*, and *Salmonella *[20].

Principles of managing these attacks in the resource limited setting include using a systematic survey to rule out major traumatic injury; once these injuries have been addressed, then focus turns to soft tissue and prevention of local and/or systemic infection. This is achieved through careful cleaning with soap and water and an anti-infective such as betadine. Tetanus and rabies vaccines also should be administered to patients who suffered unprovoked attack from any wild animal. Prophylactic antibiotics are used in our setting, though they remain controversial in general. One study has proven that post-bite infection may be reduced to < 2.0% in domestic cat and dog bites when prophylactic antibiotics are used, and suggests that antibiotics may be prudent in wild animal attacks [21]. Further argument for prophylactic antibiotics in our setting include the following: the rural location of many attacks, poor transport systems, and subsequent late presentation of injuries; puncture-type wounds; and, high rate of immunodeficiency in the East African population [22]. Regarding the surgical management of these wounds, it is most ideal to attempt primary closure of facial injuries for cosmetic purposes. However, in the clinical setting of immunodeficiency or high risk for infection in a cat, dog, monkey, or livestock wound, we emphasize that delayed primary closure represents the most appropriate surgical management [23].

## Conclusion

With trauma triage of animal attacks; vaccination against viruses and antibiotic prophylaxis against common animal-borne organisms in the initial period after attack; and, appropriate surgical management, wild animal injuries can be managed effectively in a resource-limited setting. Given the increasing human-wild animal encounters in changing ecosystems and increasing population in East Africa, rural and tertiary care providers should be familiar with the triage and treatment of varying animal attacks, and when these require referral or can be managed remotely. Further, as attacks potentially increase in the future due to human population burden, community awareness initiatives directed toward both prevention and appropriate treatment of these attacks may be warranted.

### Consent

Written informed consent was obtained from the parent of the 6 year old and other patients.

## Conflict of interests

The authors declare that they have no competing interests.

## Authors' contributions

KM (Manuscript writing, collection of data), VK (study idea, collection of data, conceptual revisions of manuscript), CA (study idea, advising, revision) All Authors read and approved the final version of the manuscript.
